# Impact of gene alterations on clinical outcome in young adults with myelodysplastic syndromes

**DOI:** 10.1038/s41598-023-29794-4

**Published:** 2023-02-14

**Authors:** Tatsuya Konishi, Daichi Sadato, Takashi Toya, Chizuko Hirama, Yuya Kishida, Akihito Nagata, Yuta Yamada, Naoki Shingai, Hiroaki Shimizu, Yuho Najima, Takeshi Kobayashi, Kyoko Haraguchi, Yoshiki Okuyama, Hironori Harada, Kazuteru Ohashi, Yuka Harada, Noriko Doki

**Affiliations:** 1 Hematology Division, Tokyo Metropolitan Cancer and Infectious Diseases Center, Komagome Hospital, 3-18-22 Honkomagome, Bunkyo-Ku, Tokyo, 113-8677 Japan; 2 Clinical Research Support Center, Tokyo Metropolitan Cancer and Infectious Diseases Center, Komagome Hospital, Tokyo, Japan; 3 Division of Transfusion and Cell Therapy, Tokyo Metropolitan Cancer and Infectious Diseases Center, Komagome Hospital, Tokyo, Japan; 4 Laboratory of Oncology, Tokyo University of Pharmacy and Life Sciences, Tokyo, Japan

**Keywords:** Genetics research, Outcomes research

## Abstract

Young adults with myelodysplastic syndrome (MDS) are rare, and the clinical significance of driver mutations has not yet been analysed. We analysed the gene mutations and copy number alterations (CNAs) in younger MDS patients using next-generation sequencing, targeting 68 genes that were recurrently mutated in myeloid malignancies, to investigate the correlation between their genetic alterations and clinical outcomes. We enrolled 55 patients retrospectively (aged < 50 years). At least one mutation was detected in 56% of the patients. The most frequently mutated genes were *ASXL1* and *RUNX1,* 13% each. We defined higher-risk patients as those with ≥ 2 mutations, except for *SF3B1* mutation, and/or CNA. The 3-year overall survival (OS) in patients with a higher-risk was lower than that in those with a lower-risk (50.8% vs. 71.8%, *P* = 0.024). Among the 44 transplant recipients, patients with higher-risk had a significantly lower OS and tended to have a higher cumulative incidence of relapse (CIR) than those with a lower-risk (3-year OS: 38.0% vs. 64.4%, *P* = 0.039; 3-year CIR: 44.0% vs. 24.1%, *P* = 0.076). Our results showed that genetic aberrations can predict clinical outcomes in younger MDS patients, despite the low rate of genetic mutations.

## Introduction

Myelodysplastic syndromes (MDS) are a heterogeneous group of clonal hematopoietic stem cell diseases characterised by ineffective hematopoiesis, peripheral blood cytopenia, and an increased risk of progression to acute myeloid leukemia^1,2^. The median age of patients with MDS is ≥ 70 years, mainly due to the accumulation of gene mutations in the hematopoietic stem cells with aging. In contrast, less than 10% of patients with MDS are < 50 years^3–5^. Therefore, limited reports exist on the clinical course and prognosis of MDS aged under 50 years, including adolescents and young adults (AYAs) aged 15–39 years. In clinical practice, treatment strategies for patients with MDS younger than 50 years of age are similar to those for patients older than 50 years old^1,2,4,6^. However, as Grabska et al. reported that among patients with MDS, AYA patients had more circulating myeloblasts and more hypoplastic MDS compared with patients older than 40 years old^6^, AYA MDS might have distinct clinical characteristics.

Genome profile analysis in MDS has progressed dramatically in the past decade with the advent of next-generation sequencing (NGS) testing, and is expected to help predict treatment response and prognosis^7–10^. MDS in older patients typically presents with clonal evolution being driven by the age-related acquisition of somatic mutations. An average of 9.2 mutations per person have been detected by whole exome sequencing^11^. Moreover, most cases appear to harbour one or more mutations^10^. Conversely, genetic predispositions play an important role in the pathogenesis of pediatric MDS (younger than 15 years) since the genetic mutations accumulation is lower in pediatric patients with MDS than that in elderly patients aged 65 years or older^9,12,13^. Therefore, myeloid neoplasms with germline predispositions have been categorized as separate entities^14^. However, reports of genetic analyses focusing on this age group are sparse^15–17^. Furthermore, chromosome abnormalities also present similar problems.

Allogeneic hematopoietic stem cell transplantation (HSCT) is the only curative treatment option for MDS patients. Given the patient’s age, comorbidities, and long-term prognosis, allogeneic HSCT is more likely to be performed in younger patients with MDS^18^. The elucidation of the correlation between the clinical outcomes, including transplant outcomes and gene mutations, will help provide better treatment strategies for this population. However, owing to the rarity of the relevant population, the association between the clinical outcomes of MDS patients aged ≤ 50 years and their gene alterations has not been clarified. Therefore, in this study, we investigated the characteristics of MDS gene mutations in younger patients using targeted sequencing and assessed their association with allogeneic HSCT outcomes.

## Materials and methods

### Patient selection

Between January 2005 and July 2018, all the admitted patients aged < 50 years at our institution were enrolled in this study if they were diagnosed with MDS or acute myeloid leukemia evolving from MDS. The final follow-up date was 20 March, 2019. Among them, we analysed patients with available NGS test results obtained using bone marrow samples preserved at the time of diagnosis.

### Definitions

We defined AYA as patients aged 15–39 years at the time of diagnosis based on the NCCN guidelines^19^ and classified MDS according to the French-American-British (FAB) criteria^20^ and the revised 2016 WHO classification. Disease risk groups and cytogenetic risks were stratified based on the International Prognostic Scoring System (IPSS) score and IPSS-R^21,22^. A complex karyotype was defined as a karyotype with ≥ 3 chromosome abnormalities. Higher-risk was defined as patients with at least one of the following characteristics; (1) more than one gene mutation, except for the *SF3B1* mutation, and (2) gene copy number alteration (CNA). Patients who were not at higher-risk were considered to be at lower-risk. Red blood cells and platelet transfusion dependence was defined as the continuous requirement of one or more transfusions every month by patients. Overall survival (OS) was defined as the time from diagnosis in the overall patient population analysis and transplantation in the HSCT recipients analysis until death from any cause, or was censored at the last follow-up. Disease-free survival (DFS) was calculated from the date of transplant to the date of death from any cause or disease progression. Cumulative incidence of relapse (CIR) and non-relapse mortality (NRM) were defined as the date of transplant to the date of disease relapse and the date of death without relapse, respectively.

### Transplantation procedures

Allogeneic HSCT was basically implemented in patients whose IPSS was intermediate-2 or high risk and/or IPSS-R was intermediate, high, or very high risk. In addition, even when IPSS was low or intermediate-1 and IPSS-R was very low or low, transfusion-dependence and/or recurrent infections could be indication for HSCT based on the previous study^23^. The patient’s comorbidities, donor availability and intention were also considered to determine the feasibility of HSCT. We selected the donor based on a standard algorithm. Specifically, an HLA-matched sibling donor was a primary choice, and if an HLA-matched donor was not available, an HLA-mismatched donor including cord blood was selected. Forty-four patients underwent allogeneic HSCT during the study period. The transplantation procedures have been described in detail previously^24^. The myeloablative conditioning regimens included cyclophosphamide (CY, 60 mg/kg for 2 days) with intravenous busulfan (ivBU, 3.2 mg/kg for 4 days) (ivBU/CY; n = 35) or total body irradiation (TBI, 12 Gy) (CY/TBI, n = 3). The reduced-intensity conditioning regimens comprised fludarabine (FLU, 30 mg/m^2^ for 6 days) with ivBU (3.2 mg/kg for 2 days) or melphalan (MEL, 40 mg/m^2^ for 2 days), with additional TBI (4 Gy) (FLU/BU2/TBI, n = 1; FLU/MEL/TBI, n = 4). In the haploidentical transplantation cases, the conditioning regimen included FLU (30 mg/m^2^ for 4 days), cytarabine (2 g/m^2^ for 4 days), CY (60 mg/m^2^ for 2 days), and TBI 8 Gy (n = 1). Graft-versus-host disease (GVHD) prophylaxis included a short course of methotrexate, cyclosporine, or tacrolimus. Tacrolimus, methylprednisolone, and rabbit anti-thymocyte globulin were used for GVHD prophylaxis in the haploidentical transplant recipients.

### Targeted next-generation sequencing

We performed a genetic analysis of the diagnostic bone marrow aspirates using NGS. The mononuclear cells harbouring leukemic blasts were isolated using the Ficoll density gradient. The genomic DNA was extracted from the mononuclear cells using an AllPrep kit (Qiagen, Hilden, Germany). The germline DNA was not available. Variant discovery using targeted amplicon-based NGS was performed as described previously^25^. The 68 target genes often present in the hematological malignancies are listed in Table S1. Finally, the mutations were checked manually by researchers experienced in hematological malignancies. The gene CNAs were calculated using the CNVKit^26^, with the pool of normal data constructed using karyotype- and copy number- normal samples.

### Statistical analysis

The frequencies between the two groups were compared using Fisher’s exact test. The non-normally distributed variables were compared between the two independent samples using the Mann–Whitney U test. The Kaplan–Meier method was used to assess OS and DFS. The differences in OS and DFS were assessed using the log-rank test. Gray’s test was used to assess the CIR and NRM; relapse and NRM were considered as competing risks. All the statistical tests were two-sided, with *P*-value < 0.05 considered as statistically significant. The statistical analyses were performed using the R software (R Foundation for Statistical Computing, Vienna, Austria).

### Ethics statement

All the participants provided written informed consent, and the study was conducted in accordance with the Declaration of Helsinki. This study was approved by the Institutional Review Board of the Tokyo Metropolitan Cancer and Infectious Diseases Center, Komagome Hospital (reference number: 2203).

## Results

### Patient characteristics

In total 87 patients (37 AYAs and 50 individuals in their 40s) were diagnosed with MDS. Of these, we analysed 55 patients (19 AYAs and 36 individuals in their 40s) using NGS data from the bone marrow samples available at the time of diagnosis. The patient characteristics are summarised in Table 1. The median age at diagnosis was 41 years (range, 19–49 years). None of the patients had a family history of hematological malignancies. The median follow-up of the surviving patients was 5.9 years (range, 0.7–13.9 years). Thirty-two patients (58%) were diagnosed with refractory anemia, and 37 (67%) had bone marrow blasts < 5% at the time of diagnosis. Twenty-three patients (41%) and 14 patients (25%) were red blood cell and platelet transfusion-dependent, respectively. The cytogenetic risk score showed that 51% of the patients were either very good or good while six cases had complex karyotypes. Monosomy 7 and t(3;3)(q21;q26), which are deemed to be cytogenetic abnormalities associated with poor-prognosis in MDS, were identified in two and one patient, respectively and all three patients were classified into higher-risk group by genetic classification. A comparison of the background between the AYAs and the patients in their 40s revealed no differences in the FAB classification or IPSS risk at diagnosis. Forty-four patients, 17 of whom were AYA patients and 27 were in their 40s, underwent allogeneic HSCT during the observation period (Table 2). Patients in the lower-risk group were more likely to have a higher hematopoietic cell transplantation-specific comorbidity index and undergo HLA-matched transplantation compared to those in the higher-risk group. Four patients in the higher-risk group received single unit cord blood transplantation. The proportion of patients with bone marrow blasts greater than 5% at the time of transplantation and patients with the history of treatment with hypomethylating agents prior to HSCT tended to be higher in the higher-risk group than in the lower-risk group. There were no differences in the other transplantation settings including the history of cytotoxic chemotherapy prior to HSCT, conditioning regimen and intensity, donor source, and interval period from diagnosis to HSCT, IPSS/IPSS-R risk at diagnosis, and age at the time of transplantation between the lower- and higher-risk groups. All the patients with complex karyotypes were included in the higher-risk group by genetic classification.

**Table 1:** Patient characteristics.

Factor	AYA (N = 19)	40s (N = 36)	*P*-value
Age at diagnosis, median (range)	31 (19–39)	46 (40–49)	< 0.001
Sex (male), n (%)	10 (52.6)	22 (61.1)	0.577
FAB classification, n (%)			0.165
RA	12 (63.2)	20 (55.6)	
RARS	2 (10.5)	0 (0)	
RAEB	3 (15.8)	8 (22.2)	
RAEB-t	2 (10.5)	3 (8.3)	
Unknown/AML	0 (0)	5 (13.9)	
WHO classification, n (%)			0.066
SLD	0 (0)	2 (5.6)	
MLD	11 (57.9)	11 (30.6)	
RS-MLD	1 (5.3)	0 (0)	
EB1	2 (10.5)	4 (11.1)	
EB2	2 (10.5)	2 (5.6)	
Unknown/AML	3 (15.8)	17 (47.2)	
Cytogenetic risk, n (%)*			0.684
Very Good	0 (0)	1 (2.8)	
Good	10 (52.6)	17 (47.2)	
Intermediate	4 (21.1)	11 (30.6)	
Poor	4 (21.1)	3 (8.3)	
NA	1 (5.3)	4 (11.1)	
IPSS risk, n (%)			
Low	3 (15.8)	9 (25.0)	0.708
Int-1	10 (52.6)	12 (33.3)	
Int-2	3 (15.8)	5 (13.9)	
High	2 (10.5)	6 (16.7)	
NA	1 (5.3)	4 (11.1)	
IPSS-R risk, n (%)			0.083
Very low	2 (10.5)	6 (16.7)	
Low	2 (10.5)	10 (27.8)	
Intermediate	9 (47.4)	4 (11.1)	
High	1 (5.3)	5 (13.9)	
Very High	4 (21.1)	7 (19.4)	
NA	1 (5.3)	4 (11.1)	
Complex karyotype, n (%)	4 (21.1)	2 (5.6)	0.167
Performance status, n (%)			0.15
0	13 (68.4)	29 (80.6)	
1	4 (21.1)	7 (19.4)	
NA	2 (10.5)	0 (0)	
Hemoglobin (g/dL), median (range)	7.9 (4.4–13.0)	10.2 (4.3–14.7)	0.126
RBC dependence, n (%)	10 (52.6)	13 (36.1)	0.144
Platelet count (× 10^9^/L), median (range)	49 (11–219)	94 (5–371)	0.025
PC dependence, n (%)	5 (26.3)	9 (25.0)	0.562
Neutrophil count (/μL), median (range)	970 (50–3,340)	1,300 (120–18,440)	0.421
Blasts in PB at diagnosis, n (%)			0.18
≦3%	17 (89.5)	25 (69.4)	
> 3%	2 (10.5)	11 (30.6)	
Blast in BM at diagnosis, n (%)			0.781
≦5%	14 (73.7)	23 (63.9)	
5–10%	2 (10.5)	3 (8.3)	
> 10%	3 (15.8)	9 (25.0)	
NA	0 (0)	1 (2.8)	
Allogeneic HSCT, n (%)	17 (89.5)	27 (75.0)	0.295

*AML* acute myeloid leukemia, *AYA* adolescent and young adult, *BM* bone marrow, *EB* excess blasts, *FAB* French-American-British, *HSCT* hematopoietic stem cell transplantation, *IPSS* international prognostic scoring system, *MLD* multilineage dysplasia, *NA* not available, *PB* peripheral blood, *PC* platelet concentrate, *RA* refractory anemia, *RAEB* refractory anemia with excess blasts, *RARS* refractory anemia with ringed sideroblasts, *RBC* red blood cells, *RS* ringed sideroblasts, *SLD* single lineage dysplasia.

*Cytogenetic risks were stratified based on the Revised International Prognostic Scoring System (IPSS-R) score^22^.

**Table 2:** Transplant recipient characteristics.

Factor	Lower-risk (N = 21)	Higher-risk (N = 23)	*P*-value
Age at HSCT, median (range)	42 (22–49)	44 (19–50)	0.182
Sex (male), n (%)	11 (52.4)	14 (60.9)	0.761
FAB classification, n (%)			0.152
RA	15 (71.4)	8 (34.8)	
RARS	1 (4.8)	1 (4.3)	
RAEB	3 (14.3)	6 (26.1)	
RAEB-t	1 (4.8)	4 (17.4)	
Unknown/AML	1 (4.8)	4 (17.4)	
WHO classification, n (%)			0.147
SLD	0 (0.0)	2 (8.7)	
MLD	12 (57.1)	5 (21.7)	
RS-MLD	0 (0.0)	1 (4.3)	
EB1	2 (9.5)	3 (13.0)	
EB2	2 (9.5)	2 (8.7)	
Unknown/AML	5 (23.8)	10 (43.5)	
Cytogenetic risk, n (%)*			0.03
Very Good	0 (0.0)	1 (4.3)	
Good	13 (61.9)	8 (34.8)	
Intermediate	7 (33.3)	5 (21.7)	
Poor	0 (0.0)	6 (26.1)	
NA	1 (4.8)	3 (13.0)	
IPSS risk, n (%)			0.451
Low	4 (19.0)	4 (17.4)	
Int-1	11 (52.4)	7 (30.4)	
Int-2	3 (14.3)	3 (13.0)	
High	2 (9.5)	6 (26.1)	
NA	1 (4.8)	3 (13.0)	
IPSS-R risk, n (%)			0.281
Very low	3 (14.3)	2 (8.7)	
Low	3 (14.3)	3 (14.3)	
Intermediate	5 (23.8)	4 (17.4)	
High	7 (33.3)	3 (14.3)	
Very High	2 (9.5)	8 (34.8)	
NA	1 (4.8)	3 (14.3)	
Complex karyotype, n (%)	0 (0.0)	4 (17.4)	0.109
RBC dependence, n (%)	9 (42.9)	12 (52.2)	0.65
PC dependence, n (%)	5 (23.8)	8 (34.8)	0.518
Performance status at HSCT, n (%)			0.736
0	11 (52.4)	9 (42.9)	
1	9 (42.9)	9 (42.9)	
≧2	1 (4.8)	3 (14.3)	
HCT-CI, n (%)			0.037
0	12 (57.1)	17 (73.9)	
1 or 2	2 (9.5)	5 (21.7)	
≧3	7 (33.3)	1 (4.3)	
HMA treatment before HSCT, n (%)	2 (9.5)	8 (34.8)	0.072
Cytotoxic chemotherapy before HSCT, n (%)	9 (42.9)	11 (47.8)	0.771
Blast in BM at HSCT, n (%)			0.068
< 5%	13 (61.9)	7 (30.4)	
≧ 5%	8 (38.1)	16 (69.6)	
Conditioning regimen, n (%)			1.000
BU/CY	18 (85.7)	17 (73.9)	
CY/TBI 12 Gy	1 (4.8)	2 (8.7)	
FLU/BU/TBI 4 Gy	0 (0.0)	1 (4.3)	
FLU/MEL/TBI 4 Gy	2 (9.5)	2 (8.7)	
FLU/CA/CY/TBI 8 Gy	0 (0.0)	1 (4.3)	
Donor source			0.095
HLA-matched related	6 (28.6)	3 (13.0)	
HLA-mismatched related	3 (14.3)	2 (8.7)	
HLA-matched unrelated	9 (42.9)	6 (26.1)	
HLA-mismatched unrelated	3 (14.3)	8 (34.8)	
Cord blood	0 (0.0)	4 (17.4)	
Graft type, n (%)			0.186
Bone marrow	16 (76.2)	14 (60.9)	
Peripheral blood	5 (23.8)	5 (21.7)	
Cord blood	0 (0.0)	4 (17.4)	
Interval from diagnosis to HSCT, n (%)			0.752
< 100 days	6 (28.6)	8 (34.8)	
≧100 days	15 (71.4)	15 (65.2)	

*AML* acute myeloid leukemia, *BM* bone marrow, *BU* busulfan, *CA* cytarabine, *CY* cyclophosphamide, *EB* excess blasts, *FAB* French-American-British, *FLU* fludarabine, *HCT-CI* hematopoietic cell transplantation-specific comorbidity index, *HMA* hypomethylating agents, *HLA* human leukocyte antigen, *HSCT* hematopoietic stem cell transplantation, *IPSS* international prognostic scoring system, *MEL* melphalan, *MLD* multilineage dysplasia, *NA* not available, *PC* platelet concentrate, *RA* refractory anemia, *RAEB* refractory anemia with excess blasts, *RARS* refractory anemia with ringed sideroblasts, *RBC* red blood cells, *RS* ringed sideroblasts, *SLD* single lineage dysplasia, *TBI* total body irradiation.

*Cytogenetic risks were stratified based on the Revised International Prognostic Scoring System (IPSS-R) score^22^.

### Distribution of gene mutations

**Figure 1:**
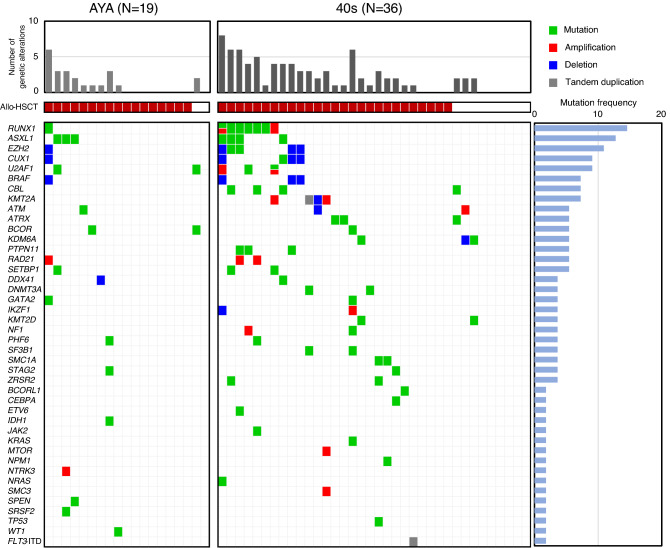
The number of genetic alterations in all 55 patients with targeted sequencing. The total number of genetic alterations in individual cases (top) in the AYA generation and in patients in their 40s, whether or not they had undergone HSCT during the clinical course (middle), and the profiles of each gene (bottom). AYA, adolescents, and young adults.

At least one driver mutation was detected in 31 of the 55 patients (56%) (Fig. 1). The most frequently mutated genes, *ASXL1* and *RUNX1*, were detected in seven patients (13%). *CBL* and *U2AF1* mutations were detected in four patients (7%), and *ATRX*, *BCOR*, *PTPN11*, and *SETBP1* in three patients (5%). Conversely, *TP53* mutation was noted in only one patient in their 40s. Additionally, *SF3B1* mutations were detected in two patients in their 40s. Both patients were classified in the higher-risk group due to having multiple mutations except for *SF3B1* (Table S2, AYAMDS sample ID 050) and having CNA (Table S2, AYAMDS sample ID 092) and ringed sideroblasts were not evident in both. The average number of mutated genes per patient was 1.3 (range 0–6), which was similar between the AYAs and those in their 40s (0.9 [range, 0–3] and 1.5 [range, 0–6], *P* = 0.25) (Fig. S1a). The proportion of patients without any gene mutations was 52% among the AYAs and 30% among those in their 40s in the gene panel set.

### Identification of copy number alterations

We performed CNA analysis in 55 patients and obtained reliable results in 51 patients, remaining four patients had low-quality data due to low DNA quantity. In total, 35 CNAs were identified (14 amplifications and 21 deletions) in 16 patients (Fig. 1); the CNAs in six patients were assumed from their chromosomal aberrations, while the remaining 10 patients had CNAs that were not as predicted from their chromosomal results (Table S2). The CNA detection rates in the AYA patients and patients in their 40s were 16% (3 of 19 patients) and 41% (13 of 32 patients), respectively. On combining the genetic mutations and CNAs, 10 patients (18%) had both mutations, and 36 patients (65%) had either of the detected mutations. Moreover, the AYA patients tended have lower total number of alterations combined with the genetic mutations and the CNAs than the patients in their 40s (mean 1.2 [range, 0–6] and 2.2 [range, 0–8], *P* = 0.08) (Fig. S1b). The proportion of patients without gene mutations or CNAs was 47% among the AYAs and 28% for those in their 40s.

### Clinical outcomes

**Figure 2:**
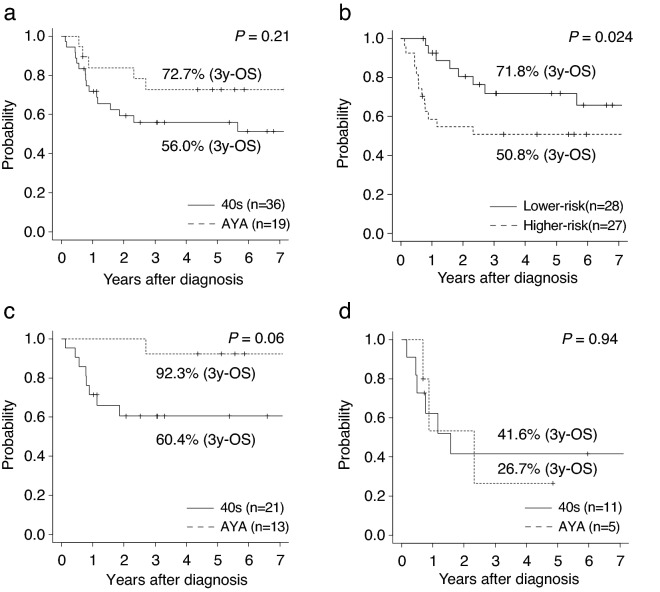
Survival outcomes of 55 patients. Overall survival (OS) compared between the AYA generation and patients in their 40s (**a**), higher-risk and lower-risk patients (higher-risk was defined as patients with two or more gene mutations and/or any copy number alterations) (**b**). OS stratified by generation in IPSS low- or intermediate-1 risk (**c**) in IPSS intermediate-2 or high risk (**d**). AYA, adolescent and young adult; CK, complex karyotype.

The 3-year OS from diagnosis in all 55 cases was 61.7% (95% CI 47.0–73.4%), 72.7% in the AYA patients (95% CI 46.3–87.6%) and 56.0% in those in their 40s (95% CI 37.8–70.9%) (*P* = 0.21) (Fig. 2a). None of the genetic mutations had a significant effect on survival. When the patients were classified according to the number of genetic mutations and CNAs, the OS was significantly higher in patients in the lower-risk group than in those in the higher-risk group (3-year OS:71.8% [95% CI 49.6–85.5%) vs. 50.8% [95% CI 30.7–67.9%), *P* = 0.024) (Fig. 2b). Furthermore, on classification by IPSS risk, the OS of AYA patients tended to be better than that of patients in their 40s in the low or intermediate-1 IPSS risk group (3-year OS:92.3% [95% CI 56.6–98.9%] vs. 60.4% [95% CI 36.0–78.0%], *P* = 0.06) (Fig. 2c). In contrast, the OS in the intermediate-2 or high IPSS risk group was comparable between the two groups (3-year OS:26.7% [95% CI 1.0–68.6%] vs. 41.6% [95% CI 13.1–68.4%], *P* = 0.94) (Fig. 2d). This trend of results was similar when classified by IPSS-R (Fig. S2).

### Allogeneic HSCT outcomes

**Figure 3:**
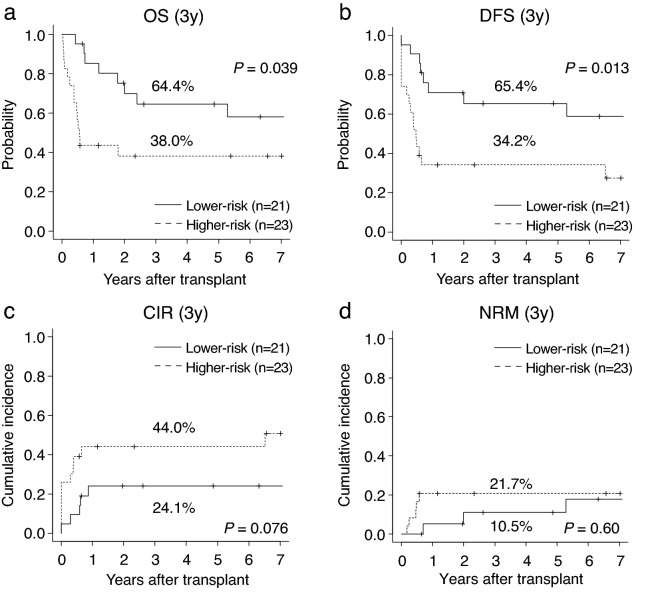
Transplant outcomes of 44 younger patients with MDS. (**a**) overall survival (OS), (**b**) disease-free survival (DFS), (**c**) cumulative incidence of relapse (CIR), and (**d**) non-relapse mortality (NRM) at 3 years after transplant were compared between the patients with higher-risk and lower-risk. CK, complex karyotype.

Regarding the clinical outcomes in 44 patients who underwent allogeneic HSCT, the OS and DFS from the day of transplantation were significantly better in the patients with lower-risk than in those with higher-risk (3-year OS:64.4% [95% CI 39.4–81.3%] vs. 38.0% [95% CI 18.6–57.4%], *P* = 0.039; 3-year DFS:65.4% [95% CI 40.6–81.8%] vs. 34.2% [95% CI 16.1–53.4%], *P* = 0.013) (Fig. 3a,b). In addition, the CIR of patients with a lower-risk tended to be lower than that of the patients with a higher-risk (3-year CIR:24.1% [95% CI 8.4–44.1%] vs. 44.0% [95% CI 22.8–63.4%], *P* = 0.076) (Fig. 3c). The NRM did not differ between the two groups (3-year NRM:10.5% [95% CI 1.6–29.2%] vs. 21.7% [95% CI 7.5–40.7%], *P* = 0.60) (Fig. 3d).

Patients who received cytotoxic chemotherapy before HSCT had significantly shorter OS from HSCT than those who were not (3-year OS:27.8% [95% CI 10.4–48.5%] vs. 69.1% [95% CI 45.7–84.0%], *P* < 0.05). On the other hand, a history of treatment with hypomethylating agents before HSCT had no impact on survival (3-year OS:50.0% [95% CI 18.4–75.3%] vs. 52.6% [95% CI 34.7–67.7%], *P* = 0.44).

## Discussion

In general, more than half of MDS patients are diagnosed at age 75 years or older, and the incidence rate of MDS in patients under 50 years of age is as low as less than 1 per 100,000)^3^. The median number of gene mutations in patients with MDS has been found to be 3–9 per individual at the time of diagnosis and most patients have more than one mutation^3,7,8,10^. In this study, we performed a genetic search of MDS patients aged < 50 years, a relatively young age group, revealed a markedly low number of mutations (1.3 per patient). Moreover, neither the genetic mutations nor the CNAs were detected in more than one-third of younger patients. In addition, the presence of multiple mutations and/or CNAs at the time of diagnosis enabled us to classify the risk groups associated with OS and DFS, and the higher-risk group tended to have a higher relapse rate after allogeneic HSCT. This was the first report to show that targeted amplicon-based NGS techniques are useful in predicting transplant outcomes in younger patients with MDS, which is consistent with the results of previous reports involving the elderly^27,28^.

Clonal hematopoiesis (CH) is a phenomenon wherein gene mutation(s) is identified in the blood cells of healthy older adults, and is associated with subsequent development of MDS and other hematological neoplasms^29,30^. Genetic mutations affecting epigenetic regulation, such as RNA splicing, DNA methylation, and chromatin modifications, are observed frequently in patients with MDS and individuals with CH^7,29^. Mutations of *ASXL1*, a gene important for epigenetic regulation, were detected in 13% of the patients in this study with the prevalence being slightly lower than that in MDS in the elderly^7^. Pediatric MDS was shown to be associated with a lower frequency of mutations in the *TET2* and *DNMT3A*, which are more frequent (approximately 10–30%) in adults. Additionally, pediatric MDS shows a higher frequency of mutations in *RUNX1*, RAS-pathway-related genes, and other germline mutations than adult MDS^9,31^. In this study of young adults, similar to the mutation profiles observed in pediatric MDS*, DNMT3A* and *TET2* mutations were rare.

Similar to our results, Mohamed et al. reported genetic mutations in AYA (18–39 years) patients with MDS with *RUNX1* mutations being most frequently detected^17^. However, *ASXL1* mutations were only detected in a small number of patients (4.6%). Recently, Epstein-Peterson et al. described the mutational profiles of 33 younger (20–50 years) patients with de novo MDS^32^. The most frequently mutated gene was *TP53* (21%), followed by *SF3B1* (18%), whereas *ASXL1* and *RUNX1* mutations were detected in only three and one patients, respectively. Our results and those of previous reports showed some discrepancies in the mutation profiles, which may have been due to the different distributions of age, disease subtypes, and ethnicity. These results suggested that MDS in young adults has intermediate characteristics between pediatric and adult MDS, although further analyses with a larger number of patients are necessary to confirm this.

MDS develops because of the accumulation of multiple gene mutations, and a high number of gene mutations is associated with poor prognosis^20,33^. Conversely, *SF3B1* mutations in MDS are considered favourable prognostic factors^34,35^. We defined the higher-risk group as patients with multiple gene mutations, excluding the *SF3B1* mutation, and/or with any CNAs. Despite no significant difference in OS according to the IPSS classification in this study, there was a difference in OS in the genetic risk stratification (Fig. 2b). Kuendgen et al. reported that the survival outcomes in MDS patients aged < 50 years were significantly better than those in older patients (median OS:176 months vs. 25 months). In particular, the OS was markedly different in patients with low- and intermediate-1 risk, whereas there was no difference according age in the intermediate-2 and high IPSS risk patients^4^. In this study, patients aged < 50 years were further divided into AYAs and those in their 40s. Similar to the previous report, the trend toward a difference in survival according to age was evident only in the IPSS low- and intermediate-1 risk group (Fig. 2c,d).

A Japanese retrospective study reported the outcomes of allogeneic HSCT in 645 patients with AYA and indicated that the 3-year OS and CIR after transplantation were 71.2% and 11.2%, respectively^36^. The OS following allogeneic HSCT in patients in the lower-risk group in our study was similar to that in the Japanese cohort, although Shimomura et al. did not evaluate the mutation profiles. Our cases included patients in their 40s and more patients with poor PS and higher hematopoietic cell transplantation-specific comorbidity index score compared with the previous report, which may have influenced transplant outcomes.

The number of gene mutations has been associated with post-transplant outcomes in adult MDS patients^28,33^, and the same correlation was observed in younger patients with MDS in this study. In analyses focused on HSCT recipients, OS and DFS were shorter, and CIR tended to be higher in the higher-risk group (Fig. 3a–c). Complex karyotype is a well-known poor prognostic factor^28^ and in this study, all the transplant patients with complex karyotypes were included in the higher-risk group by genetic classification. Although Monosomy 7 is also a common cytogenetic abnormality in MDS^2^ and was identified in two patients in this study, there did not seem to be a clear difference in the prevalence of these chromosomal abnormalities when compared to patients with MDS older than 50 years of age. Yoshizato et al. reported that *NRAS*, *TP53*, and *CBL* mutations and 17p loss of heterozygosity are strongly associated with poor prognosis in adult patients with MDS undergoing transplantation therapy^28^. These mutations were detected in a small number of young MDS patients in this study, suggesting that the acquisition of these driver mutations with age may show a deterioration in the post-transplantation outcomes, and specific prognostic indicators for younger HSCT recipients with MDS are warranted. Moreover, the presence of CNA is known to affect the clinical outcomes after transplantation, as well as the genetic mutations^28^. This study showed that the combined risk classification of genetic mutation and CNA allowed stratification of post-transplant outcomes, even in younger patients.

This study has several limitations. First, the sample size was small. However, the sample size in our study is larger than that in the previously reported studies. Second, because germline DNA was not obtained, we could not confirm whether the detected mutations were of germline origin. The VAFs of gene mutations in seven patients including one in the AYA group and six in their 40s were close to 50% or 100%, suggesting a possible germline origin. These possible germline mutations might affect the clinical outcomes after HSCT, although the exact impact of these predispositions is largely unknown^37^. Transplantation from a donor with a predisposition to myeloid malignancy should be avoided because of the risk of donor cell leukemia^37^. However, most of the patients in our study underwent unrelated HSCT. Third, targeted sequencing could not be used to identify gene mutations outside the panel. The gene panel used in this study primarily detect somatic mutations pathognomonic in adult MDS, and some important genes specific for pediatric MDS and/or inherited bone marrow failure syndromes were not included. The low number of detected mutations in our study may have been due to unidentified pathogenesis specific for younger MDS patients, and the use of whole genome/exome sequencing in more patients may be useful to detect pathognomonic markers.

In conclusion, despite the low rate of genetic mutations in younger patients with MDS, our results showed that clinical outcomes can be predicted using mutation profiles obtained with NGS techniques. Additional analyses in other cohorts are warranted to further improve the treatment strategies for this population.

## Supplementary Information

Supplementary Figures.

Supplementary Tables.

## Data Availability

The data that support the findings of this study are available from the corresponding author upon reasonable request.
