# Supplementary material for: Impact of gene alterations on clinical outcome in young adults with myelodysplastic syndromes
Source: Sci Rep. 2023 Feb 14;13:2641. doi: 10.1038/s41598-023-29794-4 (PMC9929038; doi:10.1038/s41598-023-29794-4)
Supplement: Supplementary file 1 — Supplementary Figures. [file 41598_2023_29794_MOESM1_ESM.pdf]

Impact of gene alterations on clinical outcome in young adults with myelodysplastic syndromes

Tatsuya Konishi, Daichi Sadato, Takashi Toya, Chizuko Hirama, Yuya Kishida, Akihito Nagata, Yuta Yamada, Naoki Shingai, Hiroaki Shimizu, Yuho Najima, Takeshi Kobayashi, Kyoko Haraguchi, Yoshiki Okuyama, Hironori Harada, Kazuteru Ohashi, Yuka Harada, Noriko Doki

Supplementary information

Fig S1,2

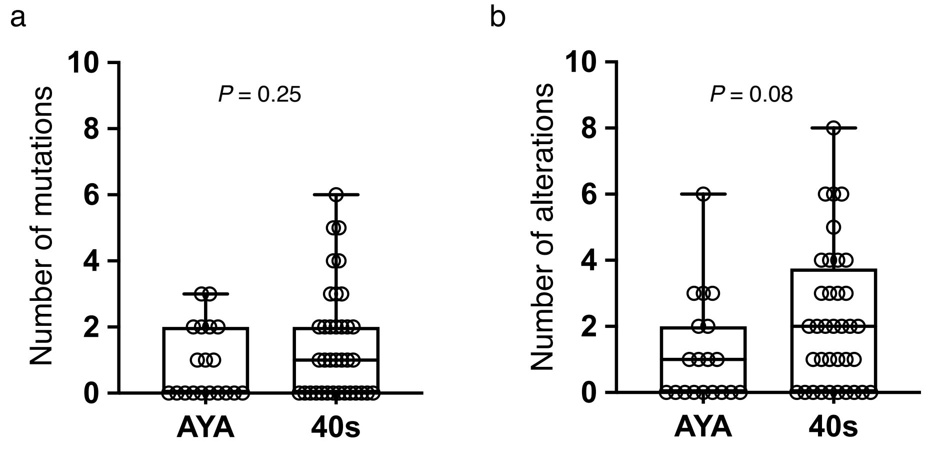

Fig. S1

Fig. S1 Number of mutated genes harboured in each case

Comparison of the number of gene mutations (**a**) and total number of gene mutations and copy number alterations (**b**) at diagnosis in patients with MDS in AYA and in their 40s. AYA, adolescents, and young adults.

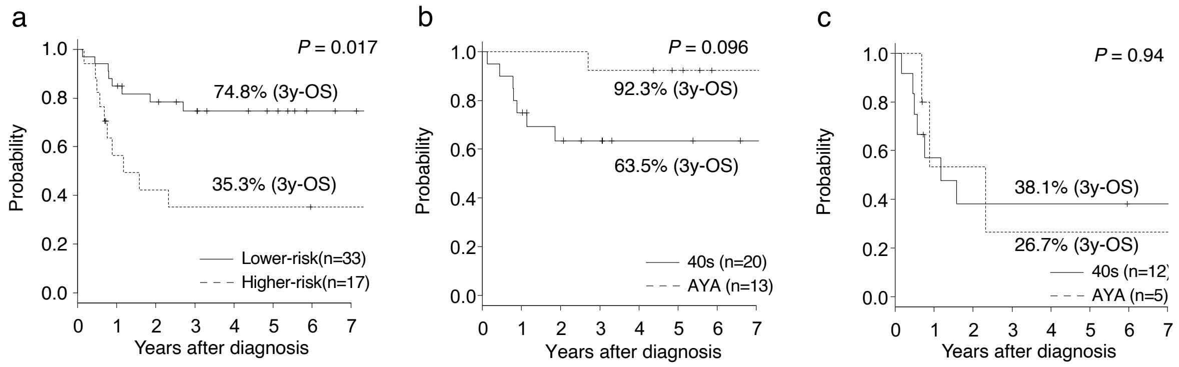

Fig. S2

Fig. S2 Survival outcomes of all the patients stratified by IPSS-R

Overall survival (OS) compared between higher-risk (high and very high risk) and lower-risk (very low, low, and intermediate risk) (**a**). OS stratified by generation in IPSS-R lower-risk (**b**) and IPSS-R higher-risk (**c**).
